# Endosomal pH, Redox Dual-Sensitive Prodrug Micelles Based on Hyaluronic Acid for Intracellular Camptothecin Delivery and Active Tumor Targeting in Cancer Therapy

**DOI:** 10.3390/pharmaceutics16101327

**Published:** 2024-10-14

**Authors:** Huiping Zhang, Liang Li, Wei Li, Hongxia Yin, Huiyun Wang, Xue Ke

**Affiliations:** 1 School of Pharmacy, China Pharmaceutical University, Nanjing 210009, China; huipingchina@yeah.net; 2 School of Pharmacy, Jining Medical College, Rizhao 276826, China; lwlw1919@163.com (W.L.); 13455018936@163.com (H.Y.); 3 Modern Tranditional Chinese Medicine Research Institute, Jiangsu Kanion Pharmaceutical Co., Ltd., Lianyungang 222000, China; liliang198761@126.com

**Keywords:** tumor targeting, pH sensitive, glutathione sensitive, camptothecin, hyaluronic acid, cancer therapy

## Abstract

**Background:** CPT is a pentacyclic monoterpene alkaloid with a wide spectrum of antitumor activity. Its clinical application is restricted due to poor water solubility, instability, and high toxicity. We developed a new kind of multifunctional micelles to improve its solubility, reduce the side effecs, and obtain enhanced antitumor effects. **Methods:** We constructed HA-CPT nano-self-assembly prodrug micelles, which combined the advantages of pH-sensitivity, redox-sensitivity, and active targeting ability to CD44 receptor-overexpressing cancer cells. To synthesize dual sensitive HA-CPT conjugates, CPT was conjugated with HA by pH-sensitive histidine (His) and redox-sensitive 3,3′-dithiodipropionic acid (DTPA). In vitro, we studied the cellular uptake and antitumor effect for tumor cell lines. In vivo, we explored the bio-distribution and antitumor effects of the micelles in HCT 116 tumor bearing nude mice. **Results:** The dual-sensitive and active targeting HA-His-ss-CPT micelles was proved to be highly efficient in CPT delivery by the in vitro cellular uptake study. The HA-His-ss-CPT micelles escaped from endosomes of tumor cells within 4 h after cellular uptake due to the proton sponge effect of the conjugating His and then quickly released CPT in the cytosol by glutathione (GSH). In mice, HA-His-ss-CPT micelles displayed efficient tumor accumulation and conspicuous inhibition of tumor growth. **Conclusions:** The novel, dual-sensitive, active targeting nano-prodrug micelles exhibited high efficiency in drug delivery and cancer therapy. This “all in one” drug delivery system can be realized in an ingenious structure and avoid intricate synthesis. This construction strategy can illume the design of nanocarriers responding to endogenous stimuli in tumors.

## 1. Introduction

Nanoparticulate drug delivery systems have been widely used in the investigation of antitumor drugs since the enhanced permeability and retention (EPR) effect was reported [1,2]. Research has indicated that nanoparticles can accumulate in fast-growing tumors due to their leaky blood vessel structure and impaired lymphatic drainage [3]. By utilizing this passive targeting effect, nanoparticles can overcome various defects of antitumor drugs, such as poor aqueous solubility and remarkable systemic toxicity [4,5]. Nanoparticles based on water-soluble polymers can prolong the drugs’ residence time in blood circulation. This is another important factor that also contributes to the improved drug accumulation in tumors [6,7]. For example, PEG-coated outside the liposomes can act as a ‘stealth’ steric barrier against the attachment of plasma protein to the liposome surface and thus reduce the clearance of liposomes from blood circulation [8].

The accumulation of nanoparticles can be further improved by the attachment of antibodies, peptides, and ligands that have a special affinity to tumors [9,10,11]. This active tumor-targeting strategy makes nanoparticles internalized into tumor cells by receptor-mediated endocytosis.

Recently, the development of stimulus-sensitive nanoparticles has become an active research area. Carefully designed nanoparticles can respond to endogenous stimuli, such as abnormal pH, high levels of glutathione (GSH), upregulated enzymes, low levels of oxygen, or reactive oxygen species (ROS) in tumors or in intracellular compartments [12,13,14,15,16]. The stimulus-sensitive nanoparticles also can respond to exogenous stimuli, such as laser irradiation, temperature changes, or magnetic fields placed near the body [17,18,19]. Among the endogenous stimulus-sensitive strategies, intracellular environment-responsive nanoparticles designed to respond to different stimuli in tumor cells can provide more effective intracellular drug delivery compared to unresponsive nanoparticles [20,21,22,23,24,25]. Further combination of two or several different responsive components that can react to different stimuli will construct multifunctional nanoparticles. The multifunctional nanoparticles can ensure precise and efficient drug release inside tumor cells and possibly provide a chance for promising antitumor compounds to realize clinical practice.

Camptothecin (CPT) is a promising compound with a wide spectrum of antitumor activity. CPT can induce tumor cell apoptosis by integrating itself into the Topoisomerase I-DNA complex and preventing DNA replication. Its application is restricted due to poor water solubility, instability, and high toxicity. CPT is a pentacyclic monoterpene alkaloid, and the active form contains an α-hydroxy lactone ring. The lactone ring is hydrolyzed under physiological pH and results in an equilibrium between the bioactive lactone form and the inactive carboxylate form [26]. In order to protect the active form of CPT from hydrolysis, we have constructed a new kind of ingenious endosomal pH, redox dual-sensitive prodrug nanoparticle. The endosomal pH, redox dual-sensitive nanoparticles may lead to thorough endosomal escape of CPT and precise drug release in tumor cells [27,28,29,30].

The pH of the tumor extracellular environment is about 6.5–7.2, lower than the pH of blood and normal tissues (pH 7.4). The pH also changes in intracellular pathways (5.0–6.0 for endosomes and 4.0–5.0 for lysosomes). Based on this extracellular and intracellular pH gradient, endosomal pH-sensitive micelles in past research have improved the intracellular delivery efficiency of antitumor drugs [30]. In addition, the glutathione (GSH) concentration in the cytosol of tumor cells is 100 to 1000 times higher than that in blood and extracellular environment. Many micelles exploiting disulfide bonds in their cores have been developed for fast intracellular release of antitumor drugs [24].

**Figure 1:**
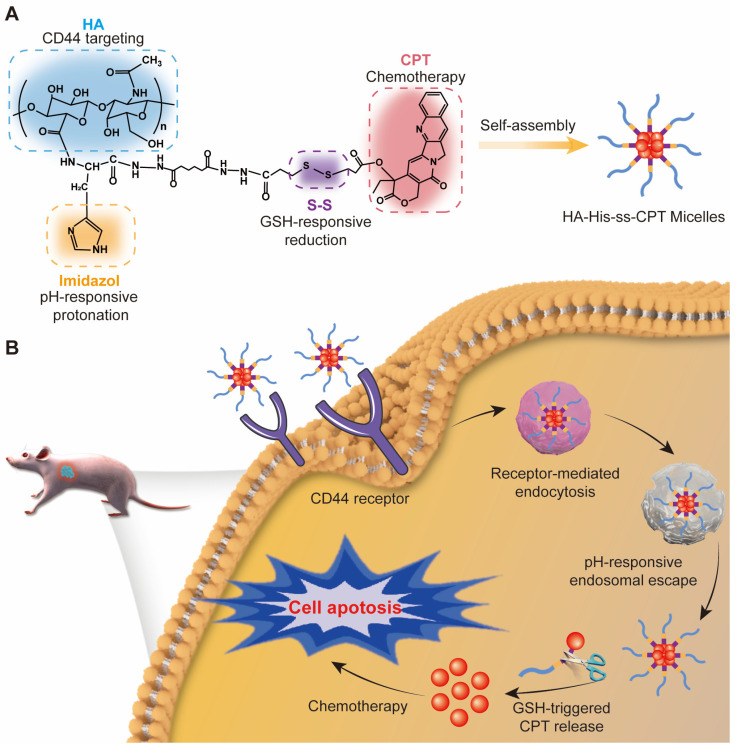
(**A**) Chemical structure of endosomal pH and redox dual-sensitive amphiphilic CPT prodrugs and construction of HHSC micelles; (**B**) schematic illustration of self-active targeting HHSC micelles for endosomal escape, GSH-triggered release of CPT and significant antitumor activity.

In our study, we chose hyaluronic acid (HA) as the carrier material and conjugated CPT to HA with histidine (His) and 3,3′-dithiodipropionic acid (DTPA) as linkers and stimuli-responsive components to build a versatile amphiphilic copolymer (Figure 1A). When in water, this copolymer would self-assemble into micelles encapsulating CPT in the inner core and keeping the lactone ring closed [31].

HA has been used in tumor-targeted delivery because of its special affinity to CD44 receptors in recent years. It also has attracted much attention for excellent physicochemical and biological properties such as biocompatibility, biodegradability, and non-immunogenicity [32]. HA of low molecular weight (LMW HA) and high molecular weight (HMW HA) have quite different CD44 receptor binding affinities (MW 5–8 < 50–60 < 175–350 kDa). However, more importantly, liposomes modified by HMW HA (175–350 kDa) displayed faster clearance compared to those modified by LMW HA (5–8, 50–60 kDa) [8]. Redox-sensitive HA-ss-paclitaxel conjugates constructed on different molecular weight HAs (MW 9.5 kDa and 35 kDa) exhibited different biological effects. HA9.5-ss-PTX displayed better cellular uptake and higher tumor-targeting efficiency than HA35-ss-PTX [33]. We chose HA of 9.5 kda as the hydrophilic macromolecules to construct a prodrug conjugate.

Histidine (His) is a classic amino acid used in the construction of pH-sensitive nanoparticles because it can make nanoparticles escape from endosomes by the proton sponge effect [34,35,36]. Its pK_a_ is 6.5, and the pK_a_ of its imidazole group is around 6. It seems that the monomer His is sensitive to tumor extracellular pH and may cause destabilization of nanoparticles. Actually, according to past research, conjugating His has a pK_a_ near the endosomal pH and has provided effective delivery of nanoparticles into tumor cells to enhance gene transfection efficiency and expression [37,38], as well as to enhance the inhibitory effect of antineoplastic drugs on tumor [39]. Thus, conjugating His that links CPT to HA should not cause the micelles to transform the shape or even disassembly in the extracellular fluid of tumor tissue. While these micelles were entrapped in endosomes after cellular endocytosis, the conjugating His can quickly absorb protons, induce an influx of counterions and water into endosomes, and eventually lead to the rupture of endosomes.

In this work, His was conjugated to HA through the amido bond. DTPA, the component providing a disulfide bond, contributed one carboxyl to conjugate with HA-His with the help of adipic dihydrazide and the other carboxyl to link with CPT. It is clear that His, DTPA, and CPT were conjugated to the repetitive disaccharide units of HA one after another in line. The biggest advantage of this linear structure was that it could ensure sufficient His in the copolymer for endosomal escape and enough CPT for the antitumor effect at the same time. It can be foreseen that this endosomal pH and redox dual-sensitive micelles would swell during endosomal escape because of proton absorption, then would be cleaved by GSH in the cytoplasm to release CPT (Figure 1B). Our results proved that the actual endosomal escape of these dual-sensitive micelles led to more effective intracellular drug delivery to the nuclei compared to the insensitive micelles. 1′-Dioctadecyl-3,3,3′,3′-tetramethylindotricarbocyanine iodide (DiR) was doped within HA-His-ss-CPT (HHSC) micelles to reveal the bio-distribution by in vivo near-infrared fluorescence (NIRF) imaging. An in vivo antitumor efficacy study evaluated the inhibition effects of HHSC micelles on tumor growth.

## 2. Materials and Methods

Sodium hyaluronate (Mw = 9.5 kDa) was kindly provided by Shandong Focusfreda Biotechnology Co., Ltd. (Qufu, China). L-histidine (His), adipic dihydrazide (ADH), 3,3′-dithiodipropionic acid (DTPA), camptothecin (CPT), succinic anhydride (SA), 1-ethyl-3-(3-(dimethylamino)propyl)1-ethyl-3-(3-(dimethylamino)propyl)carbodiimide hydrochloride (EDC), N,N′-Diisopropylcarbodiimide (DIC), N-hydroxysuccinimide (NHS), 4-dimethylaminopyridine (DMAP), gluthatione reduced form (GSH), and pyrene were purchased from Aladdin Biochem Co., Ltd. (Shanghai, China). 1′-dioctadecyl-3,3,3′,3′-tetramethylindotricarbocyanine iodide (DiR) was purchased from Dalian Meilun Biotech Co., Ltd. (Dalian, China). Lyso tracker red DND-99, Sytox, 3-(4,5-dimethylthiazol-2-yl)-2,5-diphenyltetra-zolium bromide (MTT), RPMI 1640 media and fetal bovine serum (FBS, Gibco BRL, Grand Island, NY, USA) were purchased from Jiangsu KeyGEN Biotech Co., Ltd. (Nanjing, China). Human colon cancer cells HCT116 were purchased from KeyGEN Biotech Co., Ltd. (Nanjing, China). BALB/c nude mice (6–8 weeks, 19–21 g) were purchased from Suzhou Sibeifu Biotechnology Co., Ltd. (Suzhou, China). 

### 2.1. Polymer Synthesis

**Figure 2:**
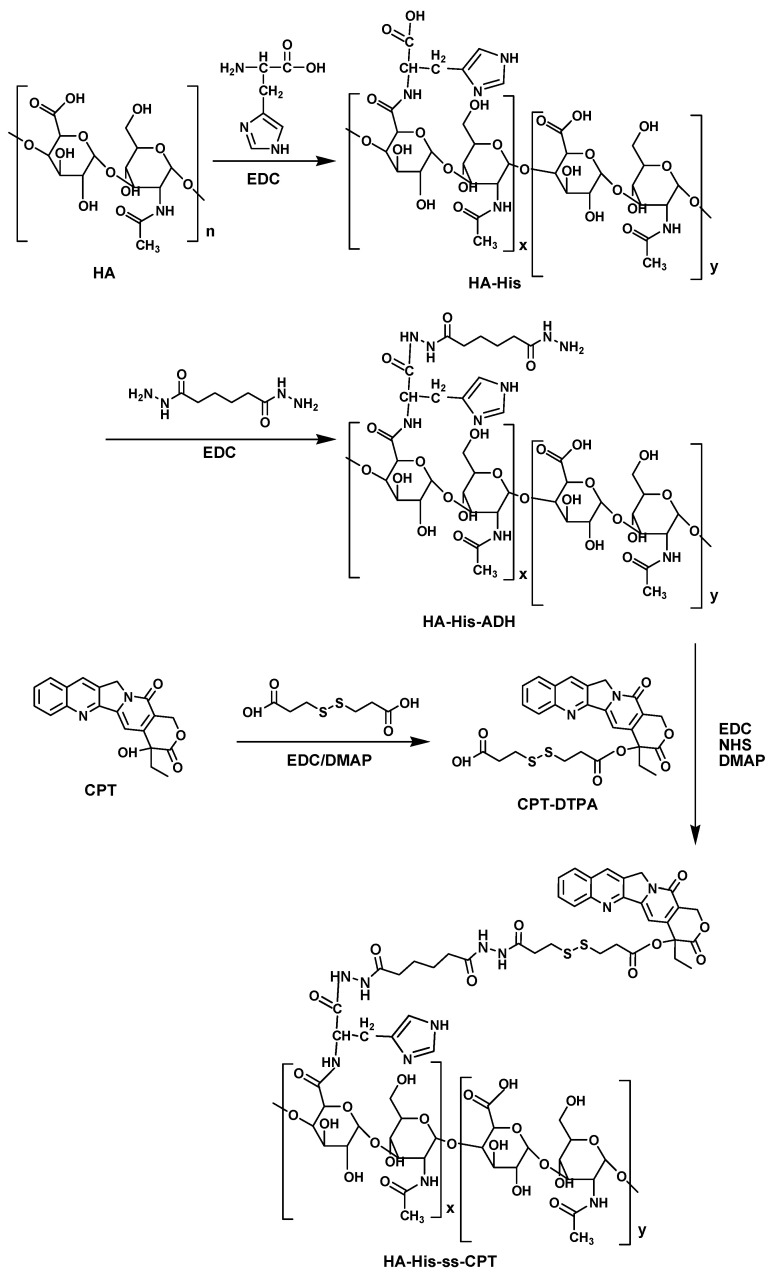
The synthesis route of HA-His-ss-CPT (HHSC).

The synthesis route of HHSC conjugate is shown in Figure 2. For comparison, the amphiphilic HA-CPT (HC) conjugate without His and disulfide linkage was also synthesized (Figure S5, Supporting Information). The structures of the copolymers were verified mainly from 1H NMR spectra recorded on a nuclear magnetic resonance spectrometer (AV300, Bruker Corporation, Fallanden, Switzerland).

*Synthesis of HA-His*. The solution of HA (200 mg, 0.5 mmol) in 10 mL water was adjusted to pH 4.75 with 0.1 M HCl [40]. Then, EDC (288 mg, 1.5 mmol) was added to the solution. The activation of carboxyl groups of HA by EDC proceeded for 30 min under stirring and was quenched by adjusting the pH to 7.0. His (465 mg, 3 mmol) dissolved in 10 mL water was then added to the mixture drop-by-drop, and the reaction lasted for 24 h at room temperature. The mixture was dialyzed against pure water for 24 h and then lyophilized to give HA-His polymer. Yield = 82%. 1H NMR (D_2_O, ppm): δ 2.12 (d, 3H, -CO-CH_3_), 2.98 (d, 2H, -CH_2_- in histidine), 7.21 (s, 1H, -N-CH=C-), 8.06 (s, 1H, -N=CH-). The substitution degree of His was calculated by the area ratio of methylene protons in His to acetyl methyl protons in HA. 

*Synthesis of HA-His-ADH.* HA-His (200 mg, 0.5 mmol) and ADH (871 mg, 5 mmol) were dissolved in 15 mL pure water, and the pH of the solution was adjusted to 4.75 with 0.1 M HCl [41]. EDC (288 mg, 1.5 mmol) was added to the solution and the reaction proceeded for 24 h at room temperature. The final solution was dialyzed against pure water for 24 h and lyophilized. Yield = 84%. 1H NMR (D_2_O, ppm): δ 1.72 (s, 4H, -CH_2_-CH_2_), 2.08 (m, 3H, -CO-CH_3_), 2.30 (s, 2H, NH_2_-NHCO-CH_2_-), 2.45 (s, 2H, -NHCO-CH_2_-), 2.96 (s, 2H, -CH_2_-in histidine). The substitution degree of ADH was calculated by the area ratio of methylene protons at 1.72 ppm in ADH to acetyl methyl protons in HA.

*Synthesis of CPT-DTPA.* Carboxyl terminal CPT-DTPA was synthesized from esterification of CPT with DTPA, which was used as a donor to introduce disulfide bond to CPT. The synthesis pathway is shown in Figure 2. In brief, CPT (174 mg, 0.5 mmol), EDC (383 mg, 2.0 mmol), DMAP (91 mg, 0.75 mmol), and DTPA (420.54 mg, 2.0 mmol) were dispersed in 250 mL CH_2_Cl_2_ [33]. The mixture was refluxed at 42 °C for 48 h. Esterification was monitored by thin-layer chromatography. The resulting solution was washed with 0.01 M HCl twice and pure water twice. Anhydrous Na_2_SO_4_ was added to adsorb the tiny water in CH_2_Cl_2_ and CPT-DTPA was given after vacuum rotary evaporation. Yield = 71%. 1H NMR (DMSO, ppm): δ 0.93 (t, 3H, -CH_3_), 2.15 (m, 2H, -CH_2_-COOH), 2.60 (m, 2H,-CH_2_-COO-), 2.96 (m, 4H, -CH_2_-S-S-CH_2_-), 3.02–3.13 (m, 2H, -CH_2_-CH_3_), 5.29 (s, 2H, -N-CH_2_-), 5.50 (s, 2H, -O-CH_2_-), 7.18 (s, 1H, =CH-), 7.71 (t, 1H, =CH-), 7.86 (t, 1H, =CH-), 8.13 (t, 2H, =CH-), 8.68 (s, 1H, =CH-). 

*Synthesis of HA-His-ss-CPT.* HA-His-ss-CPT (HHSC) conjugate was synthesized by coupling HA-His-ADH with CPT-DTPA (Figure 2). Briefly, carboxyl group of CPT-DTPA (108 mg, 0.2 mmol) was activated by EDC (191.7 mg, 1.0 mmol), NHS (115.1 mg, 1.0 mmol), and DMAP (122.2 mg, 1.0 mmol) in 12 mL anhydrous DMF at room temperature for 24 h. The activated CPT-DTPA was added dropwise to the HA-His-ADH (100 mg) in 16 mL formamide. The mixture was warmed to 40 °C for 48 h with gentle agitation. The resulting solution was dialyzed against pure water for 24 h, filtered, and lyophilized to give HA-His-ss-CPT. Yield = 79%. 1H NMR (DMSO, ppm): δ 0.92 (t, 3H, -CH_3_), 1.83 (m, 4H, -CH_2_-CH_2_-), 2.08 (s, 3H, -O-CH_3_), 2.17 (m, 2H, -NHCO-CH_2_-), 2.88 (m, 2H, -CH_2_- in histidine), 5.31 (s, 2H, -N-CH_2_-), 5.50 (s, 2H, -O-CH_2_-), 7.12 (s, 1H, =CH-), 7.72 (t, 1H, =CH-), 7.89 (t, 1H, =CH-), 8.16 (m, 1H, =CH-), 8.72 (s, 1H, =CH-).

### 2.2. Preparation and Characterization of HHSC Micelles and HC Micelles

Micelles were prepared from HHSC conjugates and HC conjugates separately by a simple ultrasonication method. Briefly, 5 mg of HHSC conjugates or HC conjugates were dissolved in 5 mL of deionized water, and the solution was sonicated at 200 w for 10 min with a probe-type ultrasonicator in ice bath. The micellar solution was filtered through 0.45 μm microfiltration membrane. The particle size and zeta potential were determined by dynamic light scattering (DLS, Zetaplus, Brookhaven Ltd., Nashua, NH, USA).

CPT content of HHSC micelles and HC micelles was measured by UV–Vis spectroscopy. The above filtrate of micellar solution was diluted with DMF to make micelles disassembled, and the absorbance of CPT in conjugate was tested at 365 nm [42]. The CPT content can be calculated using a standard curve from known concentrations of CPT solutions. 

To evaluate the change of size and zeta potential along with pH, the HHSC micelles were dispersed into PBS of different pHs (7.4, 6.5, and 5.0) and the size and zeta potential were monitored along with time. 

### 2.3. Critical Micelle Concentration (CMC) of HHSC Conjugates

The CMC of HHSC conjugates was determined by fluorescence spectroscopy using pyrene as probe. Pyrene dissolved in acetone was added into a series of volumetric flasks and then acetone was evaporated under reduced pressure. A total of 5 mL of HHSC conjugate solution at concentrations ranging from 1 × 10^−6^ mg/mL to 0.1 mg/mL were added into these volumetric flasks; meanwhile, the final concentration of pyrene was controlled to be 5 × 10^−7^ mol/L. The mixture was sonicated for 30 min at room temperature and then kept in dark for 24 h. The fluorescence spectra of the prodrug were performed with a fluorescence spectrophotometer (RF-5310 PC, Shimadzu Corporation, Kyoto, Japan) at an emission wavelength of 390 nm, and excitation spectra were recorded from 220 to 360 nm [41]. The intensity ratios of I339/I333 were plotted versus the logarithm of micellar concentrations. The CMC was estimated as the cross-point when extrapolating the intensity ratios at low- and high-concentration regions.

### 2.4. In Vitro Stability of HHSC Micelles

For investigation of in vitro stability, HHSC micelles were incubated in deionized water and RPMI 1640 medium containing 10% fetal bovine serum (FBS). The diameter and zeta potential of HHSC micelles were measured at predetermined time using DLS.

### 2.5. In Vitro Drug Release Behavior of HHSC Micelles

The reduction responsive release behavior of CPT from HHSC micelles was measured by dialysis method using PBS buffer solutions at physiological pH (pH 7.4) containing 0 μM, 10 μM, 10 mM, and 40 mM GSH separately [42]. In brief, micelle solutions were transferred into dialysis bag (MWCO 12 kda), immersed in 40 mL of release media, and kept shaking at 100 cycles/min and 37 °C. At predetermined time intervals, 3.0 mL of release media was removed and replaced with corresponding PBS containing GSH. 

PBS buffer solution at endosomal pH (pH 5.0) containing 40 mM GSH was also used as release medium to simulate the release of CPT from the swelled micelles after they had escaped from the late endosomes.

The concentration of the released CPT was detected by a fluorescence spectrophotometer at the excitation wavelength of 365 nm and the emission wavelength of 430 nm. The CPT content was calculated according to the calibration curve obtained from the known concentrations of CPT solutions.

### 2.6. Endosomal Escape of HHSC Micelles

HCT116 cells were seeded on 20 mm glass-based culture dishes at a density of 1 × 10^4^ cells/dish and incubated for 24 h. Then, the cells were treated with HHSC micelles with a CPT concentration of 10 µg/mL for 0.5 h and 4 h, respectively. After incubation, the cells were washed gently with PBS. The late endosomes were marked with Lysotracker red DND-99 at room temperature for 1 h. Then, the cells were washed with PBS and fixed with paraformaldehyde for 10 min. After that, the cell nuclei were counterstained by SYTOX at room temperature for 10 min, and then the cells were washed with serum-free medium [43]. The intracellular distribution of HHSC micelles and the late endosomes was observed by confocal laser scanning microscopy (CLSM, LSM 800, Carl Zeiss AG, Oberkochen, Germany). 

### 2.7. Intracellular Localization of HHSC Micelles 

The cell uptake and intracellular release behavior of free CPT and different micelles were evaluated by CLSM. HCT116 cells were seeded on 20 mm glass-based culture dishes at a density of 1 × 10^5^ cells/dish and incubated for 24 h. The culture medium was replaced with serum-free medium containing free CPT, HHSC micelles, and HC micelles separately at equivalent concentration of CPT (10 µg/mL) for 0.5 h and 4 h. After incubation, the cells were fixed with paraformaldehyde for 10 min and washed with serum-free medium. Then, the nuclei were stained by SYTOX at room temperature for 10 min, and the cells were washed with serum-free medium at last. The uptake and intracellular distribution of different forms were observed via CLSM.

### 2.8. In Vitro Cellular Uptake of HHSC Micelles

Quantitative determination of different CPT forms internalized into HCT116 cells was performed by flow cytometry analysis. HCT116 cells were seeded in a 6-well plate (1 × 10^5^ cells/well) and treated with free CPT and different micelles for 0.5 h and 4 h. After respective incubation, cells were rinsed twice with cold PBS and harvested using trypsin. The cells suspended in 0.5 mL cold PBS were subjected to flow cytometric analysis by a NovoCyte flow cytometer (NovoCyte 3130, ACEA Biosciences Inc., San Diego, CA, USA) to test the fluorescence intensity of CPT (405 nm) [44].

To verify that HHSC micelles had special affinity for CD44 receptors, HCT116 cells preincubated with HA (10 mg/mL) for 2.5 h were treated with the micelles for 4 h [39], and cells only treated with the micelles were used as comparison. The following procedure was just the same as above. The uptake efficiency was analyzed by the flow cytometer.

### 2.9. In Vitro Cytotoxicity Assays of HHSC Micelles

The in vitro cytotoxicity of HHSC micelles and HC micelles were assessed by MTT assay using free CPT as comparison. Briefly, HCT116 cells were seeded in 96-well plates at a density of 5.0 × 10^3^ per well and incubated for 24 h. The medium was replaced by 100 μL of cell culture medium containing HHSC micelles, HC micelles, or free CPT at various concentrations and cultured for 48 h. The cells without any treatment were used as negative controls.

Afterward, 20 μL of MTT solution (5 mg/mL in PBS) was added into each well and incubated for another 4 h at 37 °C. Then the medium containing unreacted MTT was removed carefully. For each well, 150 μL of DMSO was added to dissolve formazan crystals. The absorbance of each well was measured in a microplate reader (ELx800, BioTech, Burlington, VT, USA) at a wavelength of 570 nm to obtain the optical density (OD) value. The cell viability was calculated as (ODtreated/ODcontrol) × 100%. The IC50 values of different CPT forms were calculated using Graphpad Prism 8.0. 

### 2.10. In Vivo Near-Infrared Fluorescence Imaging

HCT116 tumor-bearing nude mice were prepared by subcutaneous inoculation with HCT116 cells (5 × 10^6^ cells/mouse) in the armpit of the mice. In vivo imaging was performed in the tumor-bearing nude mice when the tumor sizes reached 200–300 mm^3^. Mice were injected intravenously with free DiR, HHSC micelles, and HC micelles (labeled with DiR), respectively, at an equivalent dose of DiR (2 mg/kg). At 12, 24, 48, 72, and 96 h post-injection, images were taken on Carestream Image Station System (Multimodal Pro Light Source 400W, Carestream Health Inc., Rochester, NY, USA) with the excitation at 720 nm and emission at 790 nm. After 96 h post-injection, mice were sacrificed, and the tumor, heart, liver, spleen, lungs, and kidneys were excised for further ex vivo fluorescence imaging [45]. The fluorescence signals in mice at different time points were analyzed, and the mean fluorescence intensity of the tumor and each organ was compared.

### 2.11. In Vivo Antitumor Efficacy Study

BALB/c nude mice (6–8 weeks, 19–21 g) were inoculated with HCT116 cancer cells (5 × 10^6^ cells/mouse). When tumors grew to 50–100 mm^3^, mice were randomly divided into four groups (n = 5/group). Mice were injected with saline, free CPT, HHSC micelles, or HC micelles via tail vein at a dose of 5 mg/kg 5 times every other day. Tumor volume and body weight of mice were monitored every other day. The tumor volume was calculated using the equation: volume = (length × with^2^)/2. 

The body weight of mice was used as an indicator of in vivo toxicity. The mice were sacrificed after 10 days, and tumors were dissected from the animals.

## 3. Results

### 3.1. Characterization of HHSC

To conjugate CPT with water-soluble HA, His and ADH were used as linkages in sequence. The chemical structure of the conjugate was verified by the 1H NMR spectrum. In the 1H NMR spectrum of HA-His (Figure S1), the peaks at 8.06 ppm (-N=CH-) and 7.21 ppm (-N-CH=C-) belonged to the imidazole group of His, and the double peaks at 2.98 ppm were ascribed to the methylene in His. The substitution degree of His on HA was around 70% which ensured enough carboxyls for the conjugation of ADH and would provide higher pH sensitivity. When ADH was linked to HA-His, the peaks at 2.30 ppm and 2.45 ppm were nearly self-same, and their total area was equal to that of peaks at 1.72 ppm (Figure S2). The area of peak at 1.72 ppm could be used to calculate the substitution degree of ADH on HA, and the result was around 33% [41].

**Figure 3:**
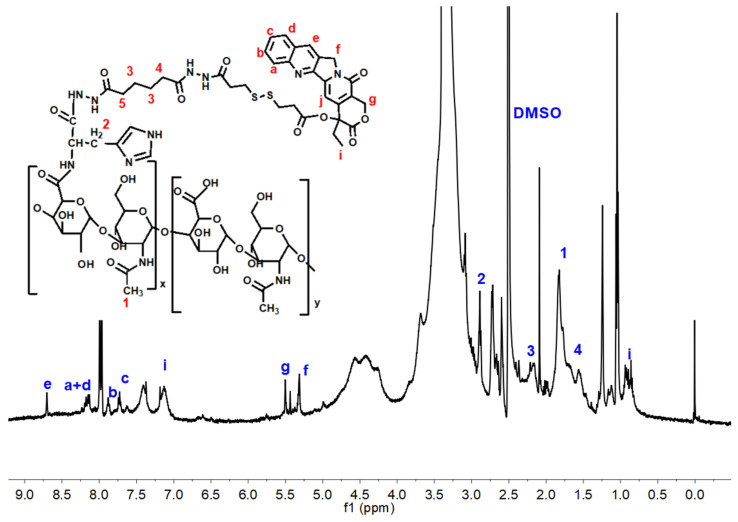
1H NMR spectrum of HHSC.

When plenty of dichloromethane was used as a solvent, one carboxyl of DTPA could be conjugated directly to CPT and another carboxyl remained unaffected. The structure of CPT-DTPA was characterized by a 1H NMR spectrum (Figure S3) and a mass spectrum (Figure S4). The 1H NMR spectrum of HHSC conjugate showed several peaks at 5–6 ppm and 7–9 ppm which belonged to the -ArH of CPT (Figure 3) [41]. These peaks proved the successful introduction of CPT-DTPA to HA-His-ADH. The characterization of HC conjugate is shown in Supplementary Materials. 

### 3.2. Preparation and Characterization of HHSC and HC Micelles

HHSC micelles and HC micelles were prepared by sonication in deionized water. As shown in Table S1, the particle sizes of both micelles were lower than 150 nm, which is of great benefit to accumulation in tumor by EPR effect. The zeta potentials of the two micelles were around −25 mV which could ensure electrostatic repulsion between micelles and dispersion stability of the micelles.

**Figure 4:**
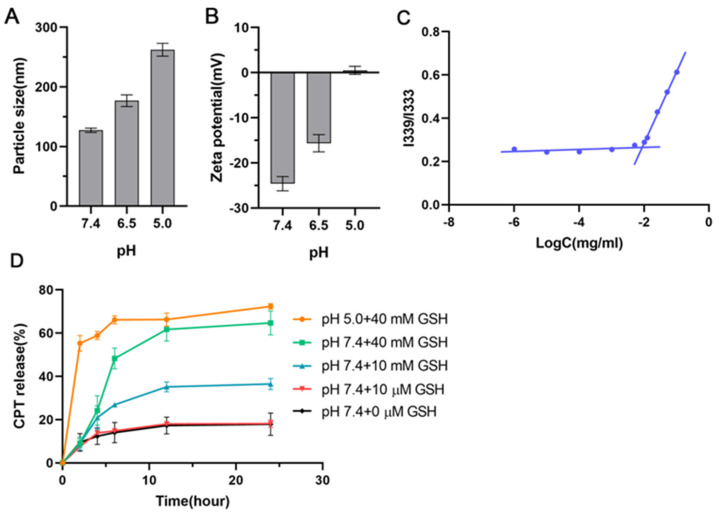
Effect of pH on particle size (**A**) and zeta potential (**B**) of HHSC micelles. All data represent mean ± SD (n = 3); (**C**) The intensity ratios I339/I333 from pyrene excitation spectra of HHSC micelles; (**D**) Percentage of CPT released from HHSC micelles in PBS buffer at pH 7.4 containing different concentrations of GSH and at pH 5.0 containing 40 mM GSH. Error bars indicate SD (n = 3).

In the study about the influence of pH on the particle size and zeta potential, HHSC micelles were expected to show a dilatation in volume and a decrease in zeta potential at acidic pH. A significant change in particle size appeared 40 min after the HHSC micelles were dispersed in PBS at pH 5.0; the particle size was doubled, as shown in Figure 4A. In contrast, there was only a relatively small increase in particle size at pH 6.5. The changes in zeta potential at pH 5.0 and pH 6.5 were both prominent. At pH 5.0, the zeta potential of HHSC micelles reversed to 0.51 ± 0.92 mV from −24.59 ± 1.58 mV (Figure 4B), which was caused by the protonation of the imidazole group in histidine. The zeta potential at pH 6.5 reduced from −24.59 ± 1.58 mV to −15.64 ± 1.91 mV, which could not be neglected but did not change the negative charge property and the function of micelles.

### 3.3. Critical Micelle Concentration (CMC) of HHSC Conjugates

In an aqueous solution, HHSC conjugates could individually self-assemble into micelles, with the hydrophobic CPT serving as the core and the hydrophilic HA part serving as the shell. Figure 4C showed the intensity ratios of I339/I333 changing against the logarithm of HHSC concentrations, and the CMC of the micelles was 8.7 µg/mL. Micelles with lower CMC value will more probably remain stable when under diluted conditions in vivo.

### 3.4. In Vitro Stability of HHSC Micelles

It is important to study the stability of HHSC micelles in deionized water and cell culture medium. DLS was used to monitor the physical characteristics of the micelles. No significant change happened in the diameter, PDI, and zeta potential of HHSC micelles incubated in deionized water for 120 h (Figures S10 and S11). The diameter of HHSC micelles incubated in RPMI 1640 medium containing 10% FBS increased slightly within 48 h and reached around 150 nm in the end (Figure S12). The fetal bovine serum contains proteins, growth factors, sugars, hormones, and enzymes. The micelles could be adsorbed to blood proteins which caused the slight increase of the diameter. The in vitro stability of HHSC micelles in 10% FBS within 48 h indicated the in vivo stability when HHSC micelles were delivered by blood to tumors. 

### 3.5. In Vitro Drug Release Behavior of HHSC Micelles

In this study, pH and GSH were used to simulate the microenvironment in the late endosome and cytoplasm of tumor cells, respectively. The pH and redox dual-sensitive HHSC micelles were hypothesized to possess better release behavior than micelles that were only designed to respond to GSH. As shown in Figure 4D, about 18 ± 1.72% of CPT was released from HHSC micelles within 24 h in PBS without or with 10 μm GSH. While at 10 mM and 40 mM GSH, 36.53 ± 2.54% and 64.68 ± 5.56% of CPT could be released from the micelles in 24 h. The results indicated that a relatively effective drug release could be obtained in the cytoplasm of tumor cells, as GSH concentration in tumor cells was about 2–10 mM [5]. At the same time, the release percentage of CPT from micelles at endosomal pH with 40 mM GSH was observed to be 55.31 ± 3.59% in 2 h, which was approximately six-fold higher than the CPT released at physiological pH with the same GSH. The accelerated release kept going on until 6 h and then slowed its velocity, but it was still faster than the release without an acidic stimulant. The micelles at endosomal pH eventually showed about 72.36 ± 1.29% of drug release in 24 h. The phenomenon revealed a clear advantage of the dual responsive HHSC micelles in the degradation of the disulfide bonds and diffusion of the loaded CPT from the inner part of micelles into the release medium, which resulted from the loose spheriform structure of micelles formed during endosomal escape.

### 3.6. Endosomal Escape of HHSC Micelles

It is believed that pH-sensitive imidazole groups of HHSC micelles can adsorb hydrions at acidic conditions of late endosomes. So, HHSC micelles will disrupt the late endosomes via the proton sponge mechanism of imidazole groups and protect themselves from degradation by enzymes in the late endosomes. Then HHSC micelles will release CPT quickly in the present of GSH in cytoplasm, and CPT will move into the nuclei. To verify this assumption, the intracellular distribution of HHSC micelles was observed.

**Figure 5:**
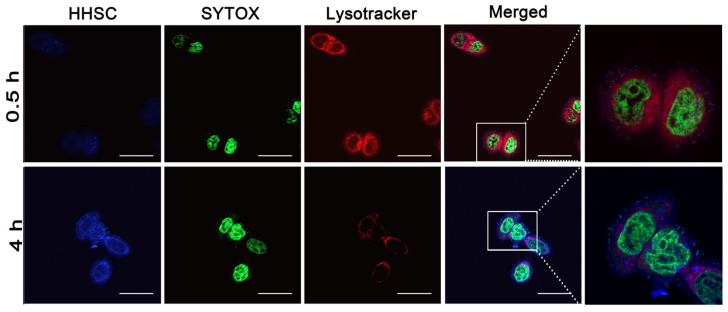
Intracellular tracking of HHSC micelles incubated with HCT116 cells for 0.5 h and 4 h. Lysotracker was used to stain the endosomes of the cell. SYTOX was used to stain the nuclei of the cell. Scale bars = 50 μm.

After an incubation of 30 min, the overlap between weak blue fluorescence signals of CPT loaded in HHSC micelles and red fluorescence signals of the late endosomes afforded rose red fluorescence signals in merged images (Figure 5). It suggested that HHSC micelles were first delivered into endosomes after being endocytosed into cells. After 4 h, strong blue fluorescence signals were observed all over the cells. It indicated that much more HHSC micelles had been internalized into cells, and a lot had escaped from the late endosomes due to their pH responsiveness. They moved across the cytoplasm releasing CPT under intracellular GSH and letting free CPT traverse into the nuclei. It was evident that HHSC micelles facilitated the endosomal escape of themselves and the internalization into nuclei of CPT.

### 3.7. Intracellular Localization of HHSC Micelles

**Figure 6:**
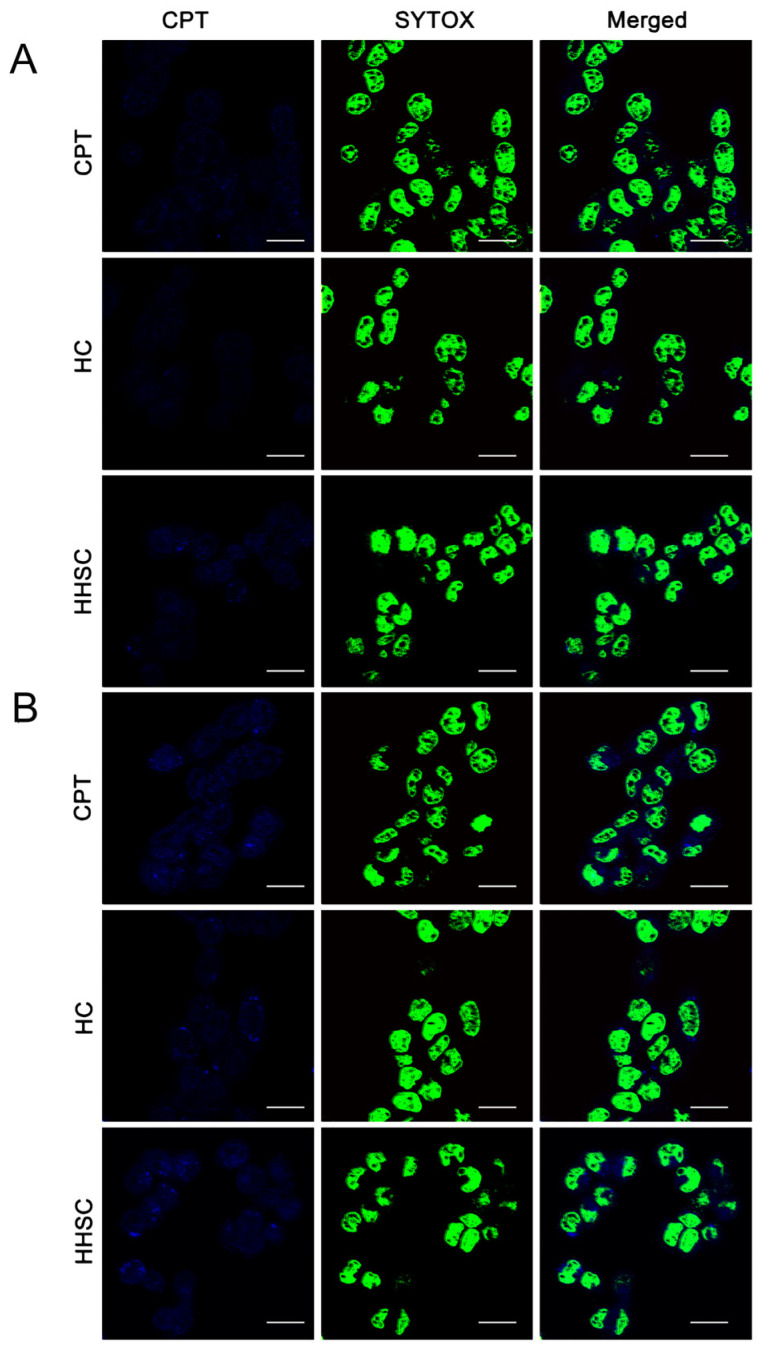
Cellular uptake and intracellular localization of HHSC micelles, HC micelles, and free CPT in HCT116 cells by CLSM at 0.5 h (**A**) and 4 h (**B**). Scale bars = 50 μm.

CLSM analysis showed the cell uptake and intracellular distribution of three different CPT forms to be time-dependent. As shown in Figure 6, the fluorescence signals of three CPT forms in HCT116 cells with an incubation of 0.5 h were much weaker than those with an incubation of 4 h. 

When cells were incubated for 0.5 h, weak blue fluorescence signals of free CPT were mainly distributed in the cytoplasm. Even weaker blue fluorescence signals of HC micelles were also observed in the cytoplasm. There were relatively strong blue fluorescence signals in cells treated with HHSC micelles, both in cytoplasm and in nuclei. It suggested that CD44 receptor-mediated endocytosis facilitated the cell uptake of HHSC micelles. The endocytosis of HHSC micelles was faster than the diffusion of free CPT within 0.5 h [46]. Endosomal escape of HHSC micelles resulted in remarkable accumulation in the cytoplasm and quick nuclear internalization of CPT released by GSH. However, much fewer HC micelles without pH responsiveness had escaped from the late endosomes, and only a little CPT appeared in the nuclei. It was obvious that the degradation of HC micelles in the late endosomes weakened the intracellular delivery so much that even free CPT accumulated more significantly in nuclei than in them. 

The fluorescence signals of HHSC micelles became further intensified after an incubation of 4 h, both in the cytoplasm and in nuclei. It suggested that the proton sponge mechanism could trigger the burst endosomal escape of these pH-responsive micelles, resulting in significant accumulation of them in the cytoplasm and rapid nuclear internalization of CPT. The fluorescence signals of free CPT looked as strong as those of HHSC micelles. The fluorescence signals of HC micelles were still the weakest among these three forms after 4 h. Focused on the nuclei in the merged photos, the fluorescence signals of HC micelles were weaker than those of HHSC micelles, which illustrated a remarkably inefficient delivery of CPT by HC micelles due to the lack of intracellular environment sensitive structural components.

As mentioned above, HHSC micelles would probably suppress the proliferation of HCT 116 cells because of their endosomal escape capacity and the advantage in the rapid release of CPT in the cytoplasm.

### 3.8. In Vitro Cellular Uptake of HHSC Micelles

**Figure 7:**
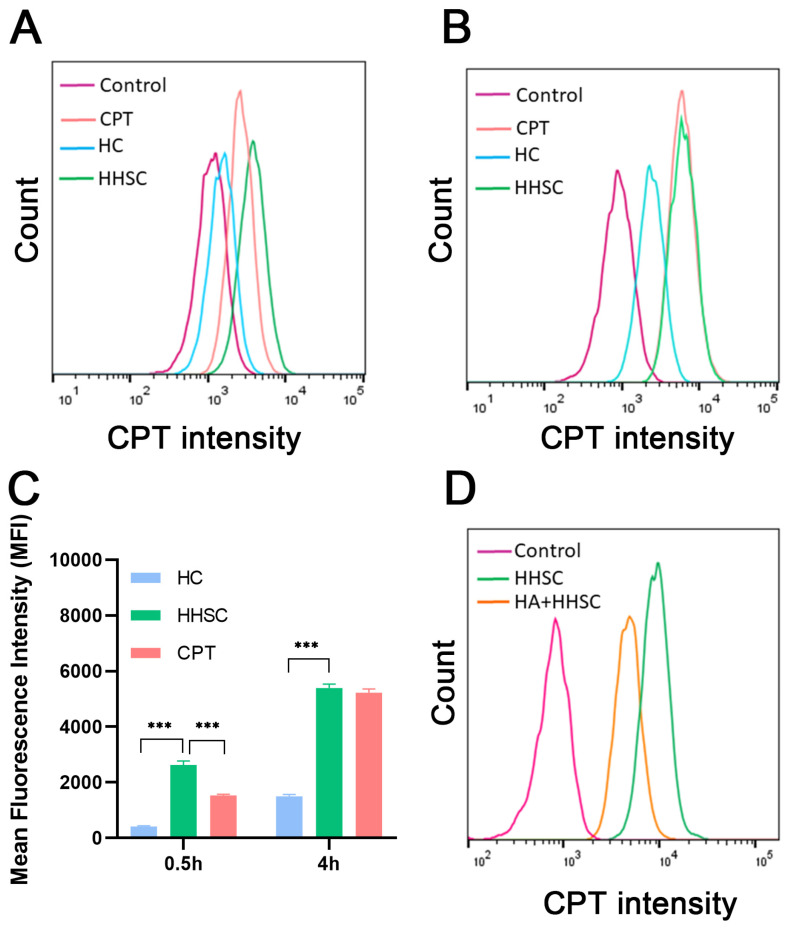
Flow cytometry profiles (**A**,**B**) and fluorescence intensity (**C**) of HCT 116 cells treated with free CPT, HC micelles, and HHSC micelles for 0.5 h and 4 h. (**D**) Fluorescence intensity of HCT 116 cells treated with HHSC micelles with or without free HA for 4 h. Error bars indicate SD (n = 3). ***: *p* < 0.001.

The cellular uptake of HHSC micelles and HC micelles, with free CPT as comparison, was further investigated quantitatively by flow cytometry, and the results were accordant with those of CLSM. The cells treated with HHSC micelles showed the highest level of CPT fluorescence signal both at 0.5 h and 4 h, and the uptake of HHSC micelles was statistically different from HC micelles (Figure 7A–C). The results confirmed that endosomal escape capacity and redox-sensitive release could enhance the intracellular delivery of HHSC micelles.

In further flow cytometry assay, the uptake of HHSC micelles by HCT116 cells pretreated with HA molecules decreased obviously (Figure 7D). The results proved that the competitive binding of free HA to CD44 receptors could inhibit CD44 receptor-mediated endocytosis of HHSC micelles. It illustrated that active cellular uptake was the internalization pathway into tumor cells of HHSC micelles.

### 3.9. In Vitro Cytotoxicity Assays of HHSC Micelles

**Figure 8:**
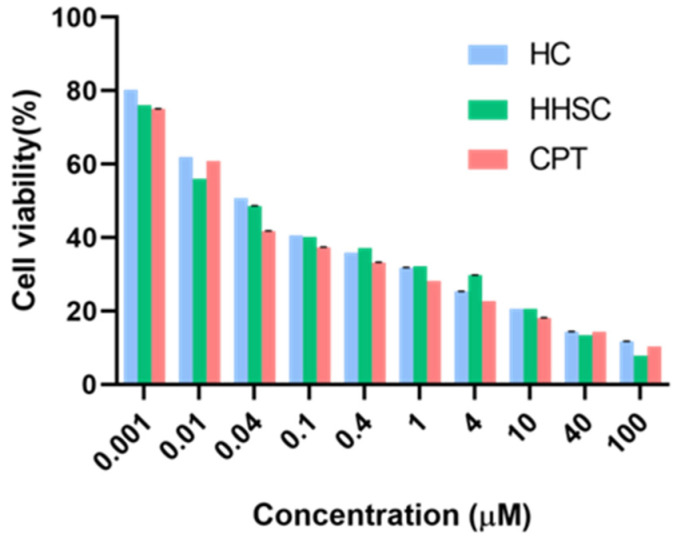
In vitro cytotoxicities of HHSC micelles, HC micelles, and CPT against HCT116 tumor cells.

The in vitro cytotoxicity of these two prodrug nanomicelles and free CPT was studied in HCT116 cells. The manifestation of HHSC micelles was comparable to that of free CPT (Figure 8). The IC50 of HHSC micelles was 0.042 μM, which was very close to that of free CPT (0.036 μM) and 28.81% lower than that of HC micelles (0.059 μM). The enhancement of cytotoxicity of HHSC micelles compared to HC micelles demonstrated the combination of pH-sensitive imidazole group and reduction-sensitive disulfide linkage to be necessary and efficient in the construction of this amphiphilic copolymer.

### 3.10. In Vivo Near-Infrared Fluorescence Imaging

Encouraged by the verification of the CD44 receptor-mediated internalization of HHSC micelles into HCT116 tumor cells, we evaluated the in vivo tumor-targeting ability of the HA-based micelles toward CD44 receptor-overexpressing solid tumors. HCT116 tumor-bearing BALB/c nude mice were intravenously injected with free DiR and DiR-labled HHSC micelles and HC micelles, respectively, followed by NIRF imaging at predetermined time intervals.

**Figure 9:**
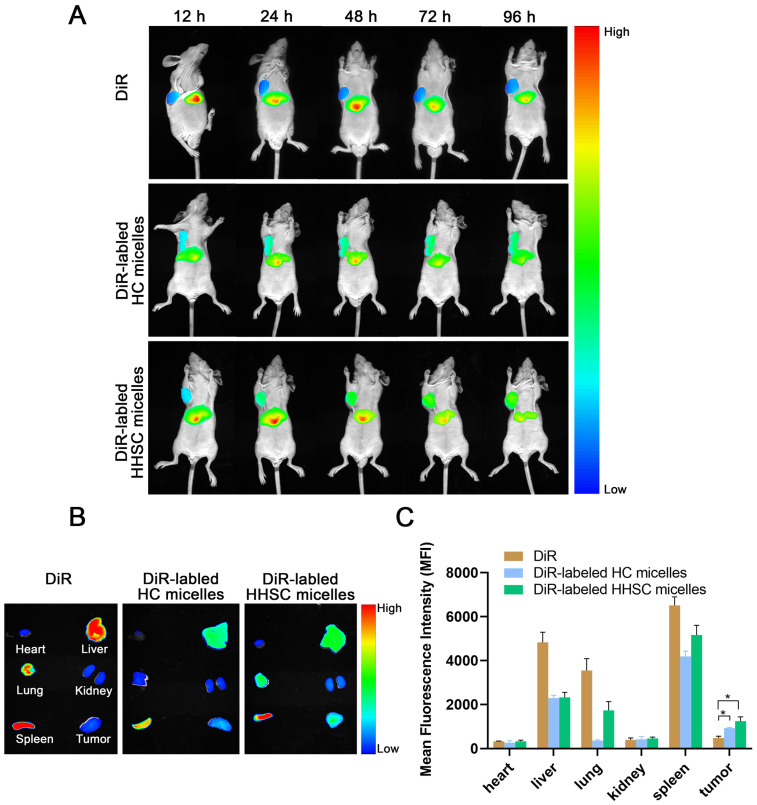
(**A**) Time-dependent in vivo NIRF images of HCT 116 tumor-bearing mice after intravenous injection of free DiR or DiR-labeled HC micelles or HHSC micelles; (**B**) ex vivo images of the main organs and tumors excised at 96 h post intravenous injection; (**C**) semi-quantitive analysis of the mean fluorescence intensity in the main organs and tumors excised at 96 h post intravenous injection. Error bars indicate SD (n = 3). *: *p* < 0.05.

As shown in Figure 9A, DiR-labled HHSC micelles accumulated effectively in the tumor at 12 h after injection, and the fluorescence signals in the tumor gradually increased along with time. The tumor accumulation of DiR-labled HC micelles along time was similar to that of DiR-labled HHSC micelles, but the fluorescence signals of HC micelles in the tumor were a little weaker than those of HHSC micelles. On the contrary, marginal tumor accumulation was observed in the group of free DiR-treated mice, though the liver uptake was almost the same as the HHSC micelles group and HC micelles group. 

Ex vivo NIRF imaging confirmed the more efficient accumulation of HHSC micelles and HC micelles over free DiR in tumors (Figure 9B). As shown in Figure 9C, the fluorescence intensity of HHSC micelles and HC micelles in tumors was 2.6 times and 1.9 times higher than that of free DiR. HHSC micelles accumulated more efficiently in tumors than HC micelles, which should be attributed to the less degradation of HHSC micelles in endosomes.

It could not be ignored that strong fluorescence signals also appeared in the spleen, liver, and lung (Figure 9B,C), which was caused by reticuloendothelial system (RES) capture due to the nanoscale characteristic of these minute prodrug micelles. But the micelles indeed relatively reduced the drug accumulation in the spleen, liver, and lung in comparison with free DiR.

In conclusion, both the in vivo and ex vivo results supported the hypothesis that HHSC micelles and HC micelles could efficiently accumulate in tumors not only by the EPR effect but also via CD44-receptor-HA-ligand interaction mediated active targeting [32,47]. 

### 3.11. In Vivo Antitumor Efficacy Study

**Figure 10:**
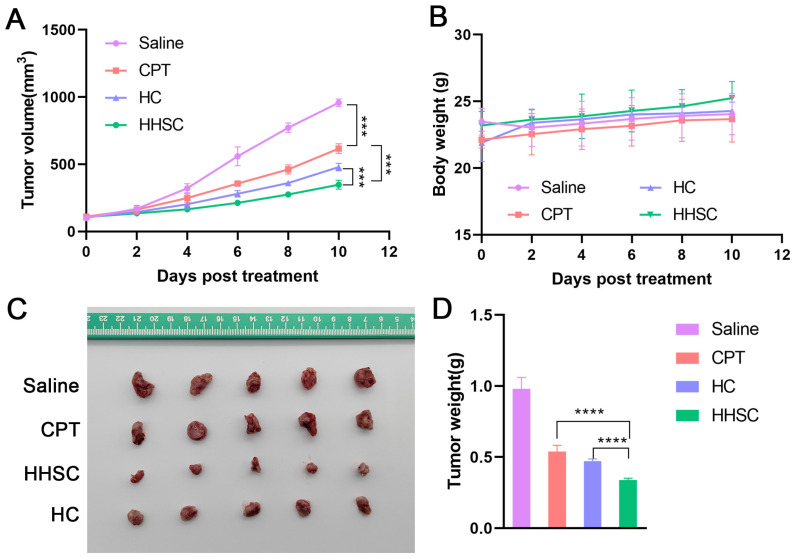
In vivo therapeutic efficacy of HCT116 tumor-bearing nude mice after intravenous injection of saline and different CPT forms at an equivalent dose of 5 mg/kg. (**A**) Tumor growth curves (***: *p* < 0.001), (**B**) body weight changes, (**C**) photos of tumors from different treatment groups excised on day 10, (**D**) tumor weights (****: *p* < 0.0001). All data represented mean ± SD (n = 5).

In vivo therapeutic effects of HHSC micelles and HC micelles were evaluated in HCT116 tumor-bearing nude mice with CPT as positive control and saline as negative control. Tumor growth was delayed in all treatment groups compared to the saline group as shown in Figure 10A. Micelles acted better than CPT in inhibiting tumor growth, which could be ascribed to EPR effects and active targeting of them. HHSC micelles suppressed tumor growth more significantly than HC micelles. 

Tumors dissected from different groups of mice are shown in Figure 10C. The mice treated with HHSC micelles had the smallest tumor volume and tumor weight (Figure 10C,D). The inhibition rate of tumor growth of HHSC micelles, HC micelles, and CPT was 65.51%, 51.84%, and 44.90%, respectively. Mice treated with different CPT forms did not show loss of body weight during the short treatment (Figure 10B). The results confirmed that HHSC micelles could enhance the antitumor activity and had slightly better safety than both HC micelles and free CPT. 

## 4. Conclusions

In this study, we developed an amphiphilic prodrug conjugate based on HA which included endosomal pH-sensitive histidines and redox-sensitive disulfide bonds. The prodrug conjugates could self-assemble into micelles in vitro with plenty of CPT in the core so as to improve the solubility of CPT. The micelles could be internalized into cancer cells via CD44 receptor-mediated endocytosis, then escape from endosomes and rapidly release CPT through breakage of disulfide linkage by GSH. The whole process ensured the highly efficient delivery of CPT to the nuclei after HHSC micelles were distributed into the tumor. In vivo imaging study proved that HHSC micelles had significant advantages in accumulation in tumors via both the EPR effect and CD44 receptor-mediated active targeting. In vivo antitumor activity study illustrated that HHSC micelles had potent efficacy in inhibiting tumor growth. HHSC micelles performed much better than HC micelles in both studies. The results confirmed the superiority of HHSC micelles in the intracellular delivery of CPT which came from the pH-sensitive and redox-sensitive components in the structure of conjugates. We could see that efficient intracellular delivery of antitumor drugs was as important as the active targeting ability. The ingenious dual-sensitive micelles we designed give enlightenment to the fabrication of new delivery systems for antitumor drugs.

## Data Availability

The raw data supporting the conclusions of this article will be made available by the authors upon request.

## Supplementary Materials

The following supporting information can be downloaded at: https://www.mdpi.com/article/10.3390/pharmaceutics16101327/s1, Reference [48] are cited in the Supplementary Materials. Figure S1: ^1^H NMR spectrum of HA-His; Figure S2: ^1^H NMR spectrum of HA-His-ADH; Figure S3: ^1^H NMR spectrum of CPT-DTPA; Figure S4: Mass spectrum of CPT-DTPA; Figure S5: The synthesis route of HA-CPT (HC); Figure S6: ^1^H NMR spectrum of HA-ADH; Figure S7: ^1^H NMR spectrum of CPT-SA; Figure S8: Mass spectrum of CPT-SA; Figure S9: ^1^H NMR spectrum of HC; Table S1: Characterization of HHSC micelles and HC micelles; Figure S10: Stability of (A) diameter and (B) zeta potential of HHSC micelles in water for 120 h; Figure S11: Stability of polydispersity index (PDI) of HHSC micelles in water for 120 h. Figure S12: Stability of diameter of HHSC micelles in RPMI1640 containing 10% FBS for 48 h.
