# Supplementary material for: Endosomal pH, Redox Dual-Sensitive Prodrug Micelles Based on Hyaluronic Acid for Intracellular Camptothecin Delivery and Active Tumor Targeting in Cancer Therapy
Source: Pharmaceutics. 2024 Oct 14;16(10):1327. doi: 10.3390/pharmaceutics16101327 (PMC11511143; doi:10.3390/pharmaceutics16101327)
Supplement: Supplementary file 1 [file pharmaceutics-16-01327-s001.zip › pharmaceutics-3175448-supplementary.pdf]

Supplementary Materials

Endosomal pH, Redox Dual-Sensitive Prodrug Micelles Based on Hyaluronic Acid for Intracellular Camptothecin Delivery and Active Tumor Targeting in Cancer Therapy

Huiping Zhang [1,2], Liang Li [3], Wei Li [2], Hongxia Yin [2], Huiyun Wang [2,*] and Xue Ke [1,*]

[1] School of Pharmacy, China Pharmaceutical University, Nanjing 210009, China; huipingchina@yeah.net
[2] School of Pharmacy, Jining Medical College, Rizhao 276826, China; lwlw1919@163.com (W.L.); 13455018936@163.com (H.Y.)
[3] Modern Tranditional Chinese Medicine Research Institute, Jiangsu Kanion Pharmaceutical Co., Ltd., Lianyungang 222000, China; liliang198761@126.com
* Correspondence: wang_huiyun@126.com (H.W.); kexue1973@vip.sina.com (X.K.)

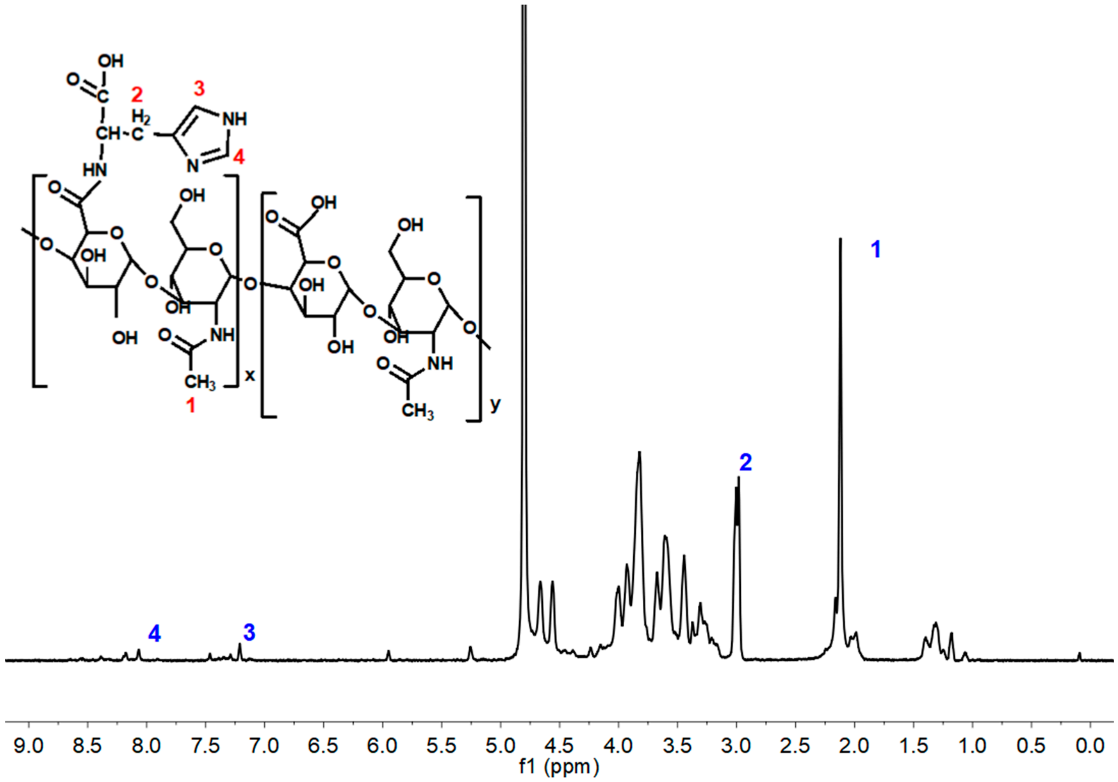

Figure S1. ^{1}H NMR spectrum of HA-His.

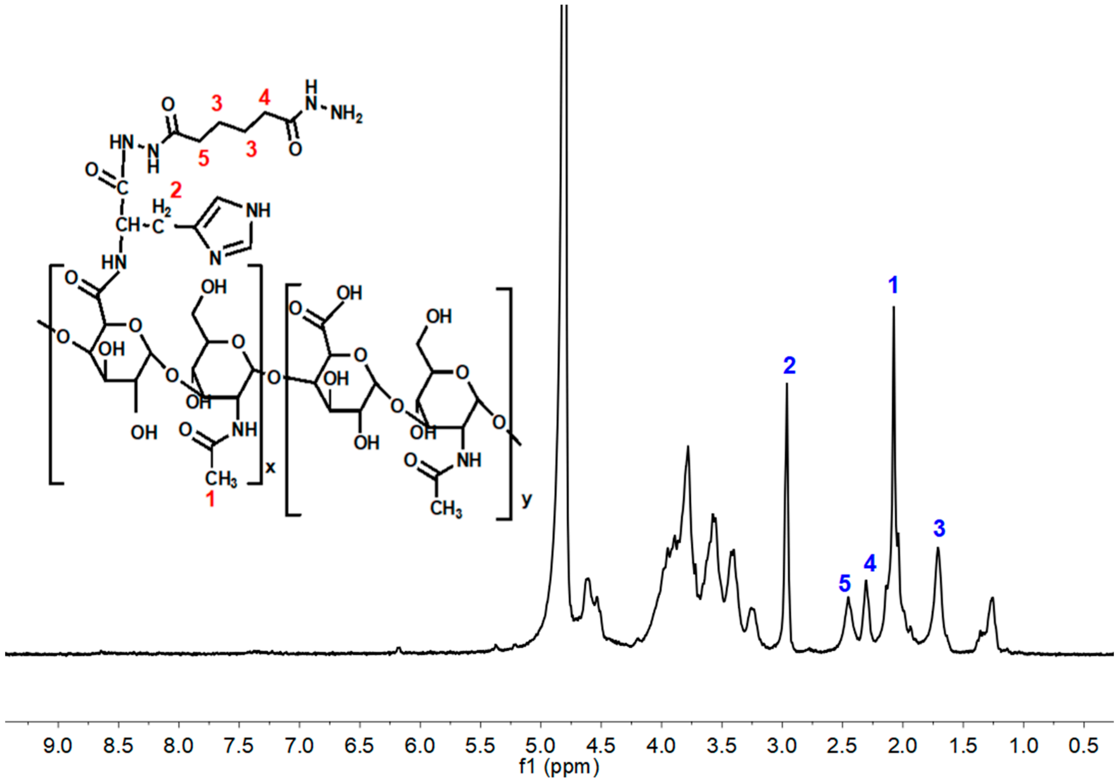

Figure S2. ^{1}H NMR spectrum of HA-His-ADH.

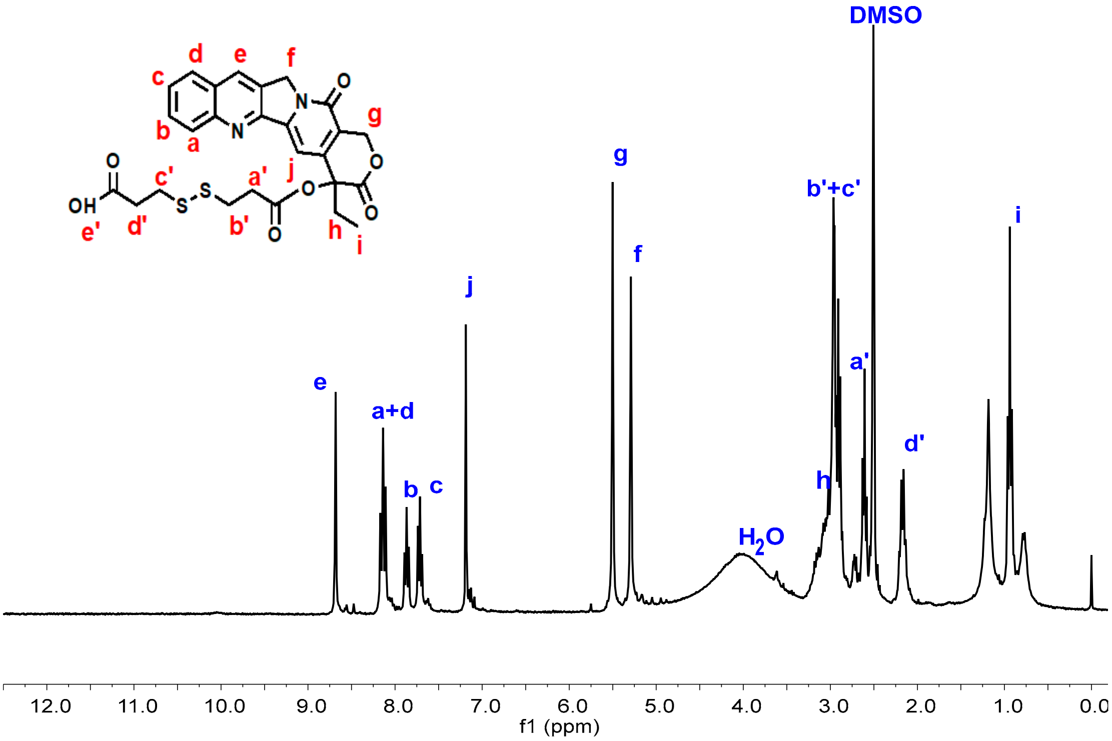

Figure S3. ^{1}H NMR spectrum of CPT-DTPA.

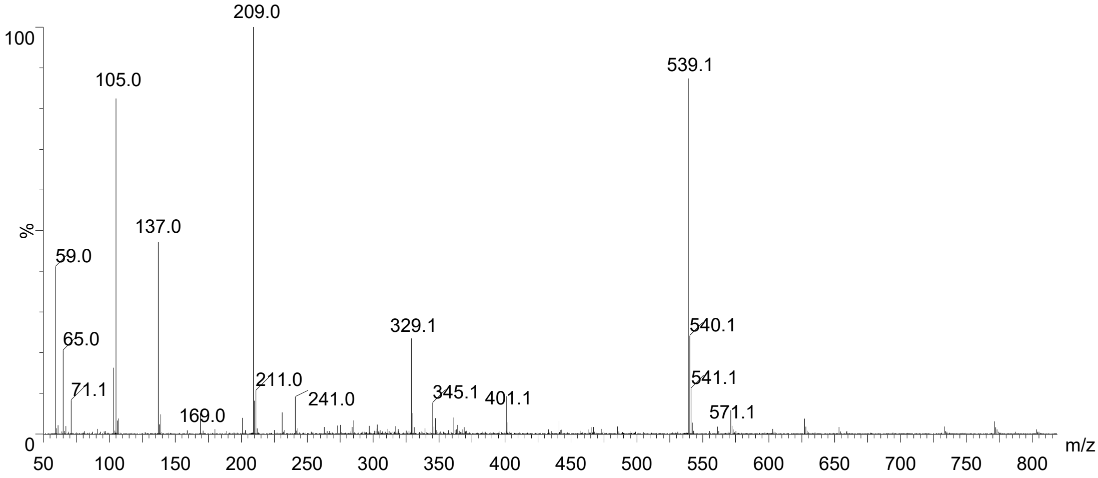

Figure S4. Mass spectrum of CPT-DTPA.

Polymer synthesis

Synthesis of HA-ADH. HA-ADH polymer was synthesized similarly as HA-His-ADH polymer. Water soluble ADH was grafted onto the backbone of HA chain using EDC in acidic solution (Figure S5). First, HA (200 mg, 0.5 mmol) and ADH (871 mg, 5 mmol) were dissolved in 15 ml pure water. Then EDC (288 mg, 1.5 mmol) was added to the solution after the pH was adjusted to 4.75. The reaction also proceeded for 24 h at room temperature. The final solution was dialyzed against pure water, filtered and lyophilized. Yield=84%. ^{1}H NMR (D_2O, ppm): δ 1.69 (s, 4H, -CH_2-CH_2-), 2.06 (t, 3H, -O-CH_3), 2.29 (s, 2H, NH_2-NHCO-CH_2-), 2.44 (s, 2H, -NHCO-CH_2-).

Synthesis of CPT-SA. Succinyloxide-modified CPT (CPT-SA) was synthesized from esterification of CPT with succinic anhydride (SA). In brief, SA (3.45 g, 34.5 mmol) and CPT (0.45 g, 1.29 mmol) were dissolved in 35 ml of dry pyridine after being stirred for 10 min at 0 ºC. A solution of DMAP (631 mg, 5.17 mmol) in 10 ml of dry pyridine was dropped into the former solution. Then the reaction proceeded for 48 h at 70 °C [48]. The reaction solution was cooled down, precipitated into excess methanol, washed with 0.5 M HCl and vacuum-dried to get dark brown CPT-SA. Yield=86%. ^{1}H NMR (DMSO, ppm): δ 0.92 (t, 3H,-CH_3), 2.16 (m, 2H,-CH_2-COO-), 2.46 (m, 2H,-CH_2-COOH), 2.75 (m, 2H,-CH_2-CH_3), 5.27 (s, 2H, -N-CH_2-), 5.48 (s, 2H,-O-CH_2-), 7.12 (s, 1H, =CH-), 7.70 (t, 1H, =CH-), 7.85 (t, 1H, =CH-), 8.09-8.18 (m, 2H, =CH-), 8.66 (s, 1H, =CH-), 12.22 (d, 1H, -COOH).

Synthesis of HA-CPT. HA-CPT conjugate was prepared by conjugating CPT-COOH to the amino groups on HA-ADH. Briefly, carboxyl groups of CPT-COOH (110 mg, 0.246 mmol) were activated by DIC (95 µl, 1.23 mmol), NHS (138 mg,1.23 mmol) and DMAP (146.4 mg, 1.23 mmol) in 10 ml of anhydrous DMF at room temperature for 24 h. Then the resulting solution of the activated CPT-COOH was added dropwise to the HA-ADH (100 mg, 0.25 mmol) in 10 ml of anhydrous formamide. The reaction proceeded at 40 ºC for 48 h. The resulting solution was dialyzed against pure water, filtered and lyophilized to give HA-CPT. Yield=80%. ^{1}H NMR (DMSO, ppm): δ 0.91 (s, 3H,-CH_3), 1.54 (s, 4H, -CH_2-CH_2-), 1.84 (s, 3H,--O-CH_3), 2.16 (s, 2H, -NHNHCO-CH_2-), 5.31 (s, 2H,-N-CH_2-), 5.48 (s, 2H,-O-CH_2-), 7.13 (s, 1H, =CH-), 7.73 (s, 1H, =CH-), 7.87 (s, 1H, =CH-), 8.15 (M, 2H, =CH-), 8.70 (s, 1H, =CH-).

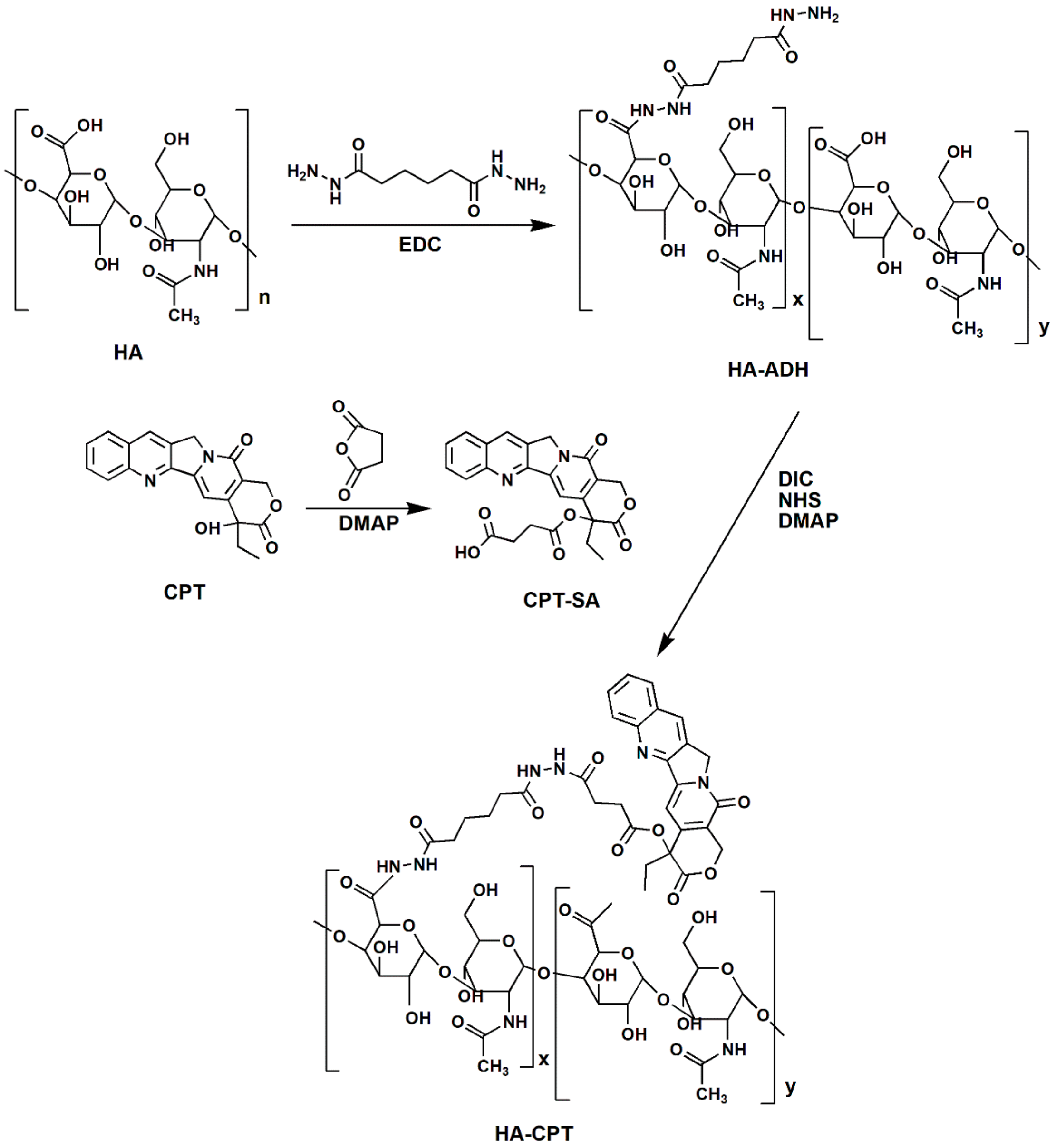

Figure S5. The synthesis route of HA-CPT (HC).

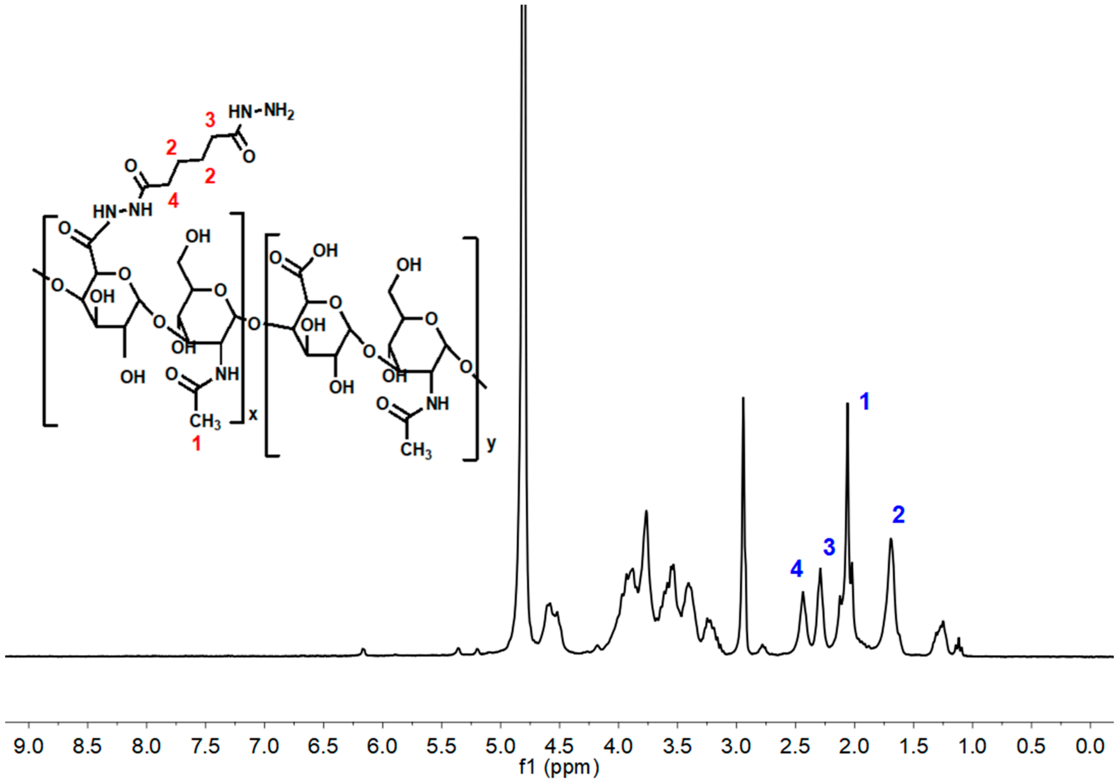

Figure S6. ^1H NMR spectrum of HA-ADH.

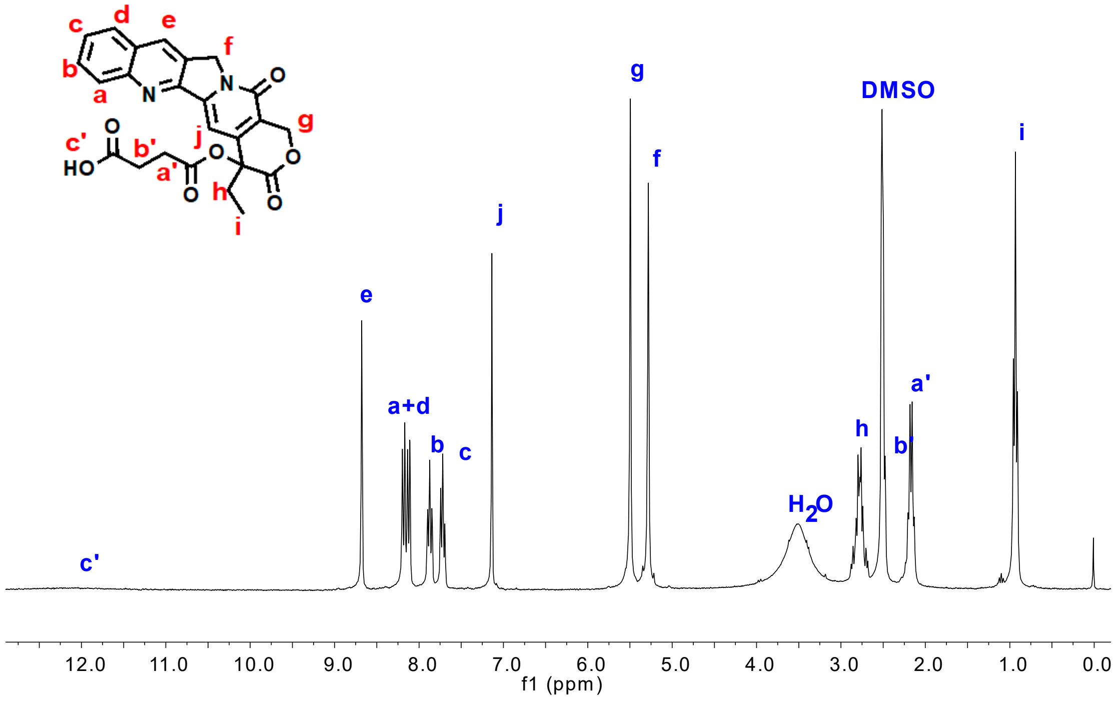

Figure S7. ^1H NMR spectrum of CPT-SA.

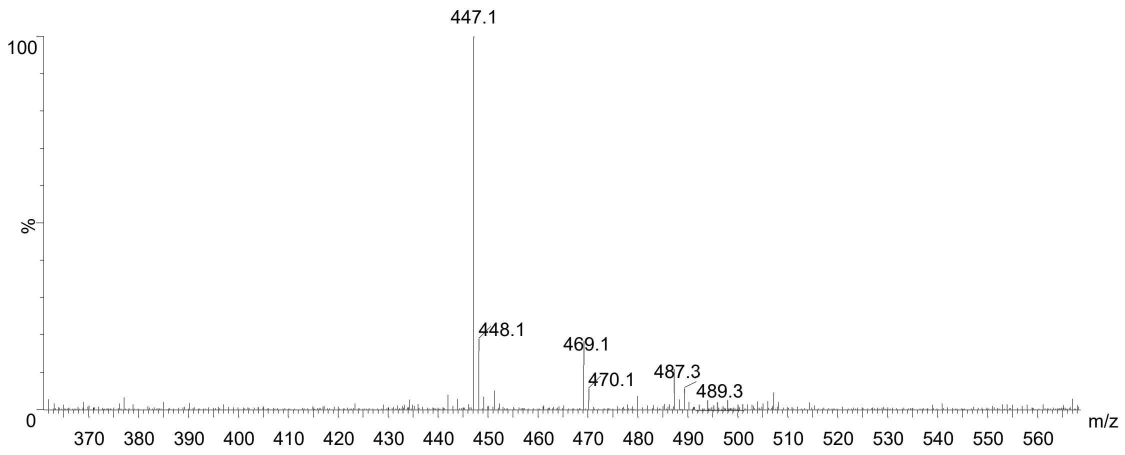

Figure S8. Mass spectrum of CPT-SA.

Characterization of HC

To conjugate CPT with water soluble HA, without endosomal pH-sensitive His and redox-sensitive disulfide bond, ADH was used as linkage as shown in Figure S5. The chemical structure of the conjugate was mainly verified by ^{1}H NMR spectrum. In the ^{1}H NMR spectrum of HA-ADH (Figure S6), the peaks at 2.29 ppm and 2.44 ppm were nearly selfsame, and the total area of them was equal to that of peaks at 1.69 ppm. The area of peak at 1.69 ppm could be used to calculate the substitution degree of ADH on HA and the result was around 51%.

The ^{1}H NMR spectrum of HC conjugate showed several peaks at 0.9 ppm, 5-6 ppm and 7-9 ppm which belonged to the -ArH of CPT (Figure S9). These peaks proved that CPT-SA had been successfully linked with HA-ADH.

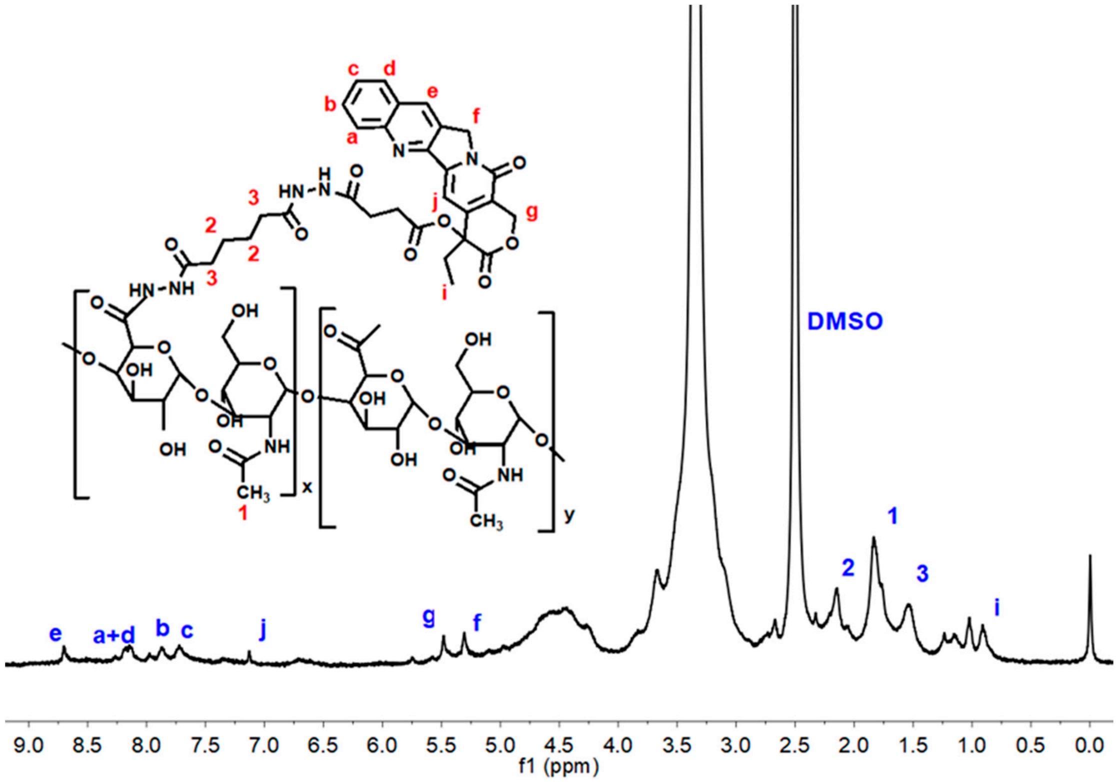

Figure S9. ^{1}H NMR spectrum of HC.

Table S1. Characterization of HHSC micelles and HC micelles (n=3).

micelles	Particle size (nm)	PDI	Zeta potential (mv)	Drug content (%w/w)
HHSC	129.1±6.1	0.252±0.011	-24.59±1.58	3.23±0.004%
HC	139.3±4.3	0.262±0.008	-26.50±3.06	4.31±0.002%

PDI-Polydispersity index.

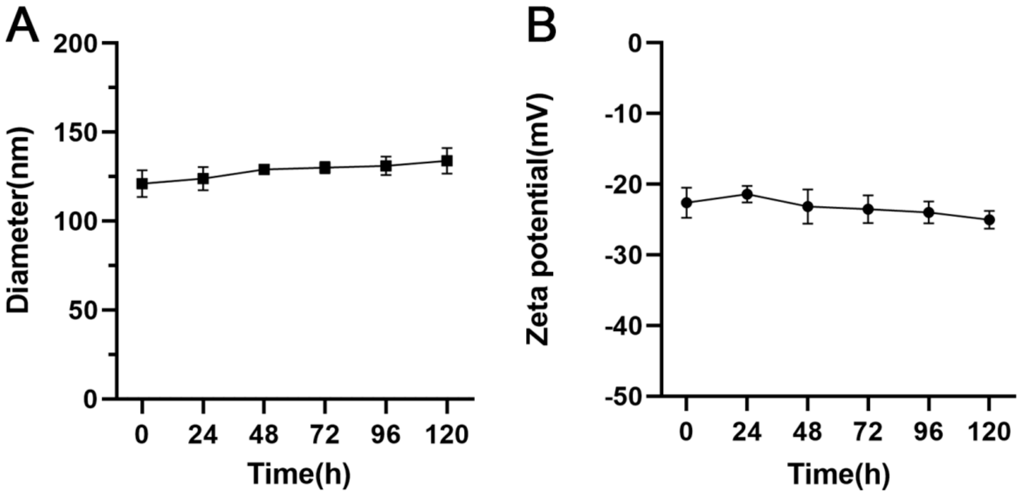

Figure S10. Stability of (**A**) diameter and (**B**) zeta potential of HHSC micelles in water for 120 h. Error bars indicate SD (n=3).

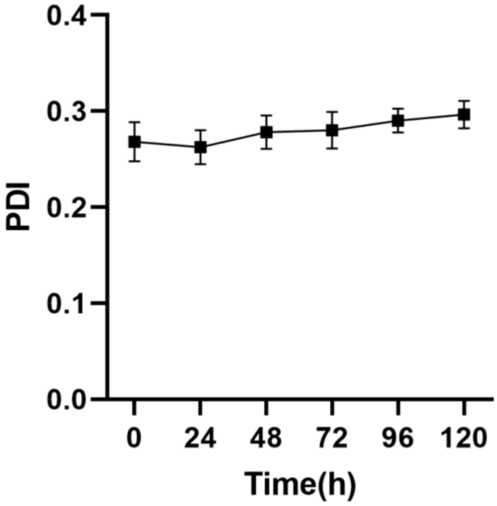

Figure S11. Stability of polydispersity index (PDI) of HHSC micelles in water for 120 h. Error bars indicate SD (n=3).

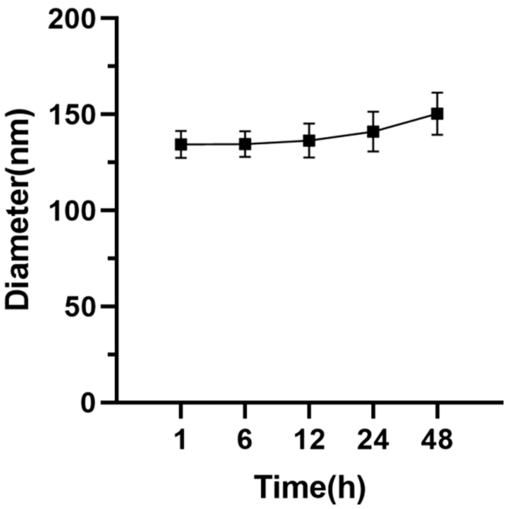

Figure S12. Stability of diameter of HHSC micelles in RPMI1640 containing 10% FBS for 48 h. Error bars indicate SD (n=3).
